# Dyssynergic patterns of defecation in constipated adolescents and young adults with anorectal malformations

**DOI:** 10.1038/s41598-020-76841-5

**Published:** 2020-11-12

**Authors:** Thomas Bjørsum-Meyer, Peter Christensen, Gunnar Baatrup, Marianne Skytte Jakobsen, Jon Asmussen, Niels Qvist

**Affiliations:** 1 Department of Surgery, Odense University Hospital, 5000 Odense C, Denmark; 2 Department of Clinical Research, Faculty of Health Science, University of Southern Denmark, 5000 Odense, Denmark; 3 Pelvic Floor Unit, Department of Surgery, Aarhus University Hospital, 8000 Aarhus, Denmark; 4 Department of Pediatrics, Odense University Hospital, 5000 Odense, Denmark; 5 Department of Radiology, Odense University Hospital, 5000 Odense, Denmark

**Keywords:** Diseases, Gastroenterology, Pathogenesis

## Abstract

We aimed to evaluate the etiologies of constipation in patients with anorectal malformations having a good prognosis for bowel control but a high risk of constipation. We included twenty-five patients from the Odense university hospital in Denmark. Patients were subjected to colon transit time examination and high resolution anorectal manometry (HRAM). The median age was 18 (14–24) and 48% (12/25) were females. Fifty-two % (13/25) of patients were diagnosed with constipation. Types of anorectal malformation were perineal fistula (9/25), rectovestibular fistula (8/25), rectourethral bulbar fistula (5/25) and no fistula (3/25). No difference in neither total colon transit time nor segmental colon transit times were found based on the presence of constipation. Only four of the constipated patients fulfilled criteria for dyssynergic defecation with a dyssynergic pattern at HRAM and prolonged colon transit time. A Type I dyssynergic pattern was dominant in constipated patients (7/13). A Dyssynergic defecation pattern was due to isolated contraction of puborectalis muscle in 9 out of 13constipated patients. We found a dyssynergic pattern during attempted defecation in patients with anorectal malformations disregarded the presence of constipation. In the majority of constipated patients an isolated contraction of the puborectalis muscle was visualized with HRAM.

## Introduction

Anorectal malformation (ARM) is an inborn anomaly affecting approximately one out of 2500 newborns^1^. Constipation is a common finding in patients operated for ARM with a reported incidence ranging from 22 to 87% at the age above 10 years^2–5^. In the general population constipation is reported in up to 30% and dyssynergic defecation (DD) is found in 25 to 50% of these^6–9^. Chronic constipation affects daily life including education and ability to work and has been associated to increased psychological distress^10^. DD is an acquired condition whereby an involuntary contraction of the external anal sphincter and puborectalis muscle may lead to fecal obstruction during defecation and in some instances overflow incontinence. The pathophysiology of constipation has been associated to prolonged recto-sigmoid transit time and the presence of DD in early childhood after operation for ARM but remains unclear in adolescence an among adults^11,12^. High Resolution Anorectal Manometry (HRAM) is a recently developed technique offering spatiotemporal plots with three-dimensional pressurization which have been used to investigate functional defecation disorders but the value compared to other diagnostic modalities is still unclear^13–16^. In a recent published study by our working group sphincter defects as detected by HRAM can be correlated to the severity of fecal incontinence but the clinical value of HRAM in evaluating constipated ARM-patients is unknown^17^.

We aimed to describe findings from colonic transit time (CTT) and HRAM in patients > 10 years of age operated for anorectal malformations having a high risk of constipation (perineal fistula, rectovestibular fistula, rectourethral bulbar fistula and no fistula) and to compare the results in ARM-patients with constipation and ARM- patients without symptoms of constipation with special regard to the incidence of signs of DD.

## Results

One hundred and twenty-seven subjects met inclusion criteria and a total of 25 (20%) subjects consented to participate. Reasons for not participating were active retrieval (n = 47) and no answer (n = 55). We were unable to perform HRAM in two patients due to anal stenosis and one patient refrained from HRAM leaving 22 patients for further analyses on HRAM.

### Demographics

Data are presented in Table 1. All patients were Caucasians, born and raised in Denmark. None of the patients were diagnosed with diabetes mellitus or other acquired metabolic or neurologic diseases known to have a possible effect on the gastrointestinal function. Cardiac anomalies were atrial septal defect (n = 9), persistent ductus arteriosus (n = 5) and ventricular septal defect (n = 3). In four patients more than one cardiac anomaly was present at birth. None of the cardiac anomalies required treatment. Renal anomaly was diagnosed in four patients with unilateral renal agenesis in threeand hydronephrosis in one. One patient (a male) who later was diagnosed with a neurogenic bladder had undergone a Mitrofanoff procedure with the creation of an appendicovesicostomy and used clean intermittent catheterization. In three patients a spinal anomaly was found. One patient had spinal filum terminal lipoma and closed spina bifida at level C6 and T12 and sacral agenesis, one patient had a tethered cord which was surgically released and one patient with an untreated syringomyelia at spinal level C7/T1. Möbius syndrome was diagnosed in one patient with facial palsy as the clinical presentation.

**Table 1:** Demographics and functional outcome, n = 25.

Parameter	
Age, years	18 (14–24)
Female sex	48 (12/25)
**Type of ARM**	
perineal fistula	9
Rectovestibular fistula	8
Rectourethral (bulbar) fistula	5
No fistula	3
Associated anomalies	52 (13/25)
**Type of repair**	
PSARP	15
Perineal	5
Dilatations	4
Cutback	1
**Functional outcome**	
Normal bowel function	12 (3/25)
**Constipation**^**a**^	52 (13/25)
Grade 1	9
Grade 2	4
Voluntary bowel movements	100 (25/25)
**Soiling**	48 (12/25)
Grade 1	7
Grade 2	4
Grade 3	1
Constipation and soiling	46 (6/13)

^a^Rome IV criteria for constipation. The severity of constipation is based on the Krickenbeck score of postoperative results. Data are presented as % (ratio) or in absolute numbers.

### Colon transit time test

Data on colon transit time is presented in Table 2. Total colon transit time and segmental transit times were all longer in patients with constipation but not at a level of statistical significance. The recto-sigmoid transit time was twice as long in patients with constipation.

**Table 2:** Results from colon transit time and high resolution anorectal manometry.

Parameter	Constipation^a^ n = 13	No constipation n = 12	P-value
Age	18 (14–24)	23 (20–24)	0.327
Female sex	6	6	1.000
BMI	20 (19–23)	22 (22–29)	0.342
Vaginal delivery	0	1	1.000
Soiling	6	6	1.000
**Colon transit time examination**			
CCT (h)	49.2 (40.8–108.0)	44.4 (25.2–61.2)	0.358
RCT (h)	18.6 (9.6–45.6)	18 (25.2–30.0)	0.704
LCT (h)	8.4 (4.8–38.4)	6 (0–41.4)	0.129
RST (h)	16.8 (12.0–21.6)	8.4 (4.2–19.2)	0.342
**HRAM**	n = 13	n = 8	
Mean anal resting pressure (mmHg)	35 (29–73)	38 (33–48)	0.889
Maximal anal resting pressure (mmHg)	43 (36–88)	50 (42–62)	0.976
Maximal anal squeeze pressure (mmHg)	124 (83–207)	96 (56–137)	0.741
Lambda configuration	6	3	0.423
Recto-anal pressure difference	− 11 (− 16 to 28)	− 11 (− 16 to 28)	0.788
Recto-anal inhibitory reflex	5	5	0.203
Anal high pressure zone (cm)	2.6 (2.5–3.2)	3.4 (3–3.7)	0.667
First sensation (ml)	40 (30–60)	40 (30–45)	0.638
Desire to defecate (ml)	60 (53–100)	50 (48–75)	0.433
Discomfort (ml)	100 (65–163)	90 (58–165)	0.535
Perineal descent	3	0	0.273
**Dyssynergic pattern**			
Type I	7	3	0.660
Type II	3	0	0.505
Type III	1	1	1.000
Type IV	2	4	0.146
Dyssynergic defecation	4	0	0.131
**Involved muscles in dyssynergic defecation pattern**			
PB	10	0	0.001*
EAS	3	2	1.000
PB + EAS	0	6	0.001*

Data are presented as medians (interquartile ranges) or in numbers.

*CCT* colonic transit time, *RCT* right colonic transit times, *LFT* left colonic transit time, *RST* rectosigmoid colonic transit time, *HRAM* high resolution anorectal manometry, *PB* puborectal muscle, *EAS* external anal sphincter.

*Statistical significant.

^a^Diagnosed by the Rome IV criteria of functional constipation.

### High Resolution Anorectal Manometry

We were not able to show any differences on HRAM parameters between groups based on the presence of constipation. A type I dyssynergic pattern was present in 54% (7/13) of patients with constipation and in 37% (3/8) of patients without constipation (Table 2). Three constipated patients showed a type II dyssynergic pattern on HRAM (21%) and one constipated patient a type III pattern (7%). In patients without constipation a type II and type III dyssynergic pattern was observed in none of the 8 patients and one out of 8 (13%) patients respectively. For type IV dyssynergic pattern, it was observed in 14% (2/14) of constipated patients and in 50% (4/8) with no constipation. In the four constipated patients meeting the diagnostic criteria for DD a paradoxical isolated contraction of the puborectal muscle was observed in two patients, isolated contraction of the external anal sphincter in one patient and simultaneously contraction of the puborectal muscle and external anal sphincter in one patient.

### Correlation analysis

Cleveland Clinic Constipation Score (CCCS) was correlated to the following HRAM parameters: mean anal resting pressure (ρ = − 0.145, p = 0.733), mean anal squeeze pressure (ρ = − 0.338, p = 0.414), recto-anal pressure gradient (ρ = 0.229, p = 0.586), first sensation (ρ = − 0.280, p = 0.503), desire to defecate (ρ = − 0.442, p = 0.272), discomfort (ρ = − 0.467, p = 0.244) and length of high pressure zone (ρ = − 0.358, p = 0.384).

CCCS was correlated to the following colonic transit time (CTT) parameters: total colon transit time (ρ = 0.128, p = 0.763), right colon transit time (ρ = − 0.108, p = 0.798), left colon transit time (ρ = 0.188, p = 0.656) and recto-sigmoid transit time (ρ = − 0.006, p = 0.989).

## Discussion

In the present study all patients fulfilling the diagnostic criteria (Rome IV) for constipation had a dyssynergic pattern at HRAM, and 69% (9/13) of these patients had a colon transit time within the normal range. Surprisingly a dyssynergic pattern was also observed in patients with no signs of constipation (8/8).

A dyssynergic pattern as detected with anorectal manometry was observed in all patients subjected to HRAM in our study without regard to the presence of constipation. Only 31% (4/13) of patients with constipation were diagnosed with DD and a type 1 dyssynergic pattern was found in all four patients.

In a retrospective study of mainly pediatric ARM-patients with constipation, dyssynergic pattern type 1 was observed in 27/28 and type IV in the last patient^12^. Information on colon transit time was not included in this study but 71% (20/28) of the patients were able to perform balloon expulsion within one minute (defecometry) and thus not fulfilling diagnostic criteria for DD. The etiology of DD is unclear. The condition may be regarded as an acquired behavioral disorder and a study found that in 2/3 of cases it was acquired during adulthood^9^. In symptomatic patients, DD is important to diagnose and treat, owing to the fact that DD is associated with impaired disease-related quality of life^18^. A dyssynergic pattern has been reported to occur in nearly 90% of asymptomatic individuals and in patients with chronic proctalgia without constipation, with high inter-observer agreement rates for type I and IV dyssynergic pattern^19,20^. This can partly be explained by a non-physiologic position during anorectal manometry as in our setting in which the patient was placed in the left decubitus position with an empty rectum. Optimal the patients should be placed on a commode for HRAM to mimic the natural defecation position but the placement of the rigid 3D-probe in the anal canal is expected to be inconvenient for examiner and elicit discomfort for the patient. Moreover the left lateral decubitus position is recommended by The International Anorectal Physiology Working Group^21^.

When it comes to treatment for DD, coaching has in one study revealed a normalization of manometric results in 12 out of 39 patients^22^. A Type I dyssynergic pattern was dominant in our patients with constipation, found in seven out of 13 cases. In a study including 40 females with constipation, a dyssynergic defecation pattern was found with HRAM in 28 (70%) with Type I in 17 (43%), Type III in 9 (23%), Type IV in 2 (5%) and none with Type II^23^. The patients were older than our population (median age 53 vs. 23 years) and previous anorectal surgery was registered in 12 (29%), not otherwise specified.

We did not find any significant differences in total or segmental colonic transit times between patients with constipation and patients without constipation. Nonetheless the recto-sigmoidal transit time was twice as long in patients reporting constipation compared to patients with no constipation. In our study the recto-anal inhibitor reflex (RAIR) was only present in 36% (5/14) of patients with constipation compared to 63% (5/8) of patients with no constipation. RAIR is characterized by transient relaxation of the internal anal sphincter in response to distension of the rectum and is an indication of functioning internal anal sphincter. Many patients with ARM lack the recto-anal inhibitory reflex probably as a consequence of corrective surgery or the inborn atresia of the anal canal. An absence of RAIR is proposed to contribute to the development of constipation as it increases anal resting pressure. In our patients with constipation RAIR was absent in 62% (8/13) suggesting that the contribution to constipation is questionable.

We found isolated paradoxical activation of the puborectalis muscle in ten out of thirteen constipated patients indicative of puborectalis syndrome. None of the patients without constipation presented isolated paradoxical puborectal activation during straining. Relaxation of the puborectalis muscle is important during attempted defecation in order to straighten the anorectal angle and facilitate the passage of stool.

Knowledge about the pathophysiology in constipated ARM-patients is important to target the treatment. An isolated slow-transit constipation benefits from treatment with laxatives but biofeedback treatment is the mainstay in constipated patients with dyssynergic defecation or absence of RAIR^24,25^. However, the evidence behind these treatments is inadequate and randomized controlled studies are needed^26^.

The strength of our study is that it was protocolized and all examination performed systematically under standardized conditions. The study also had limitations. The cohort was relative small and therefore the comparisons between even smaller groups based on the presence of constipation have limited statistical value. The unnatural left decubitus position during HRAM as mentioned previously may have overestimated the true incidence of dyssynergic defecation. We did not perform endoscopy in our population to rule out stenosis as a cause of constipation which normally is part of a diagnostic work-up. We did not perform contrast enema examinations in our patients due to ethical considerations but are aware that it would provide valuable information regarding the presence of an abundant sigmoid colon or rectum which is expected to affect colon transit times.

## Conclusion

In a long-term follow-up of patients with anorectal malformations we found that the presence of dyssynergic pattern at high resolution anorectal manometry was not related to the presence of constipation. In the majority of constipated patients an isolated contraction of the puborectalis muscle was observed.

## Methods

### Patients

We recruited patients from a tertiary pediatric surgical center at Odense University Hospital (OUH), Denmark with a covering an area of 3.5 million people. Patients with ARM were identified through local databases using the ICD-9 codes: 725.1, 725.2 (1985–1994) and ICD-10 codes: Q42 (all included) and Q438K (1995–2004). The electronical files were retrospectively reviewed. Inclusion criteria were anorectal anomalies with a good prognosis for bowel control; perineal fistula, rectobulbar urethral fistula, rectovestibular fistula and no fistula and primary treatment (surgical reconstruction or dilatations alone) for anorectal malformations from 1985 to 2004 at OUH. Exclusion criteria were cognitive disability to an extent that the patient could not properly understand purpose and consequences of the study and the presence of an enterostomy.

All patients that met inclusion criteria were sent an invitational letter and if no response was registered, another letter was sent 14 days later.

Data obtained from patient charts were: Age, comorbidity, type of malformation, type of primary surgery for anorectal malformation, and other known congenital malformations. For female patients that experienced pregnancy, we also recorded data regarding the type of delivery.

### Statistics

A non-normal distribution of data was assumed. Data were presented as medians and interquartile ranges (IQR) if not otherwise indicated. Discrete data were compared with Fisher`s exact test and for continuous data the Mann–Whitney U test was applied. Level of significance was reported with two-sided p-value with a value < 0.05 considered as statistical significant. Correlation tests were performed with the Spearmann`s rank order correlation and reported with a correlation coefficient rho (ρ) and two-sided p-value.

### Constipation diagnosis

Patients were verbally screened for functional constipation according to the Rome IV criteria (Table 3) when verbal and written informed consent was obtained for study inclusion^27^. In order to be categorized as constipated in this study, patients needed to meet the Rome IV criteria for functional constipation.

**Table 3:** Rome IV criteria for functional constipation^27^.

The following criteria should be present for at least 3 months with symptom onset at least 6 months prior to diagnosis
**1. Presence of ≥ 2 of the following symptoms:**
Lumpy or hard stools (Bristol stool form scale 1–2) in > 25% of defecations
Straining during > 25% of defecations
Sensation of incomplete evacuation for > 25% of defecations
Sensation of anorectal obstruction/blockage for > 25% of defecations
Manual maneuvers for to facilitate > 25% of defecations (digital manipulations, pelvic floor support)
< 3 spontaneous bowel movements per week
2. Loose stools rarely present without the use of laxatives
3. Insufficient criteria for irritable bowel syndrome

Content of the table is adapted from: Palsson et al.^27^.

### Questionnaires

Individual questionnaires regarding functional bowel outcome were merged in one paper document and filled in by patients before clinical examinations. The patients were asked to fill in the questionnaires by them self but were allowed to address the clinician for clarifications at the ambulatory consultation for HRAM.

#### Krickenbeck classification

The Krickenbeck classification of postoperative functional results in patients with ARM was used to evaluate bowel outcome in terms of voluntary bowel control, soiling and constipation^28^.

Krickenbeck classification of postoperative results:

Voluntary bowel movements (Feeling of urge, capacity to verbalize and able to hold bowel movements): yes/no.

##### Soiling

Grade 1: Occasionally (once or twice per week), grade 2: Every day, no social problem, grade 3: Constant, social problem.

##### Constipation

Grade 1: Manageable by changes in diet, grade 2: Requires laxatives, grade 3: Resistant to laxatives and diet.

#### Cleveland clinic constipation score (CCCS)

The severity of constipation was evaluated with CCCS (Table 4)^29^. Scores ranged from 0 (best) to 30 (worst).

**Table 4:** Cleveland clinic constipation score (CCCS).

Item	Score				
	0	1	2	3	4
Frequency of bowel movements	1–2 times/1–2 days	2X/week	1X/week	< 1X/week	< 1X/month
Painful evacuation effort	Never	Rarely	Sometimes	Usually	Always
Incomplete evacuation	Never	Rarely	Sometimes	Usually	Always
Abdominal pain	Never	Rarely	Sometimes	Usually	Always
Time for evacuation attempt/ min	< 5	5–10	10–20	20–30	> 30
Unsuccessful evacuation attempts per day	Never	1–3	3–6	6–9	> 9
Duration of constipation/years	0	1–5	5–10	10–20	> 20

The content of the table is adapted from: Agachan et al.^29^.

### Clinical examinations

#### High resolution anorectal manometry

We used a Manoscan Anorectal High Resolution Manometry system (Medtronic, MN, USA) mounted with a 3D probe. The rigid probe had 256 pressure sensors circumferentially aligned over the length of 64 mm and a diameter of 10.75 mm. The probe was calibrated before use in each participant. A disposable sheath with a rectal balloon was applied and lubricated before introducing the probe into the anus. Neither enema or colonic preparation nor sedatives were used before examination. Participants were placed in the left decubitus position with hips and knees flexed 90 degrees during the procedure. Before introducing the probe, a rectal examination was done to ensure the rectum was empty. The same clinician (TBM) with experience in performing anorectal manometries performed all examinations.

**Figure 1:**
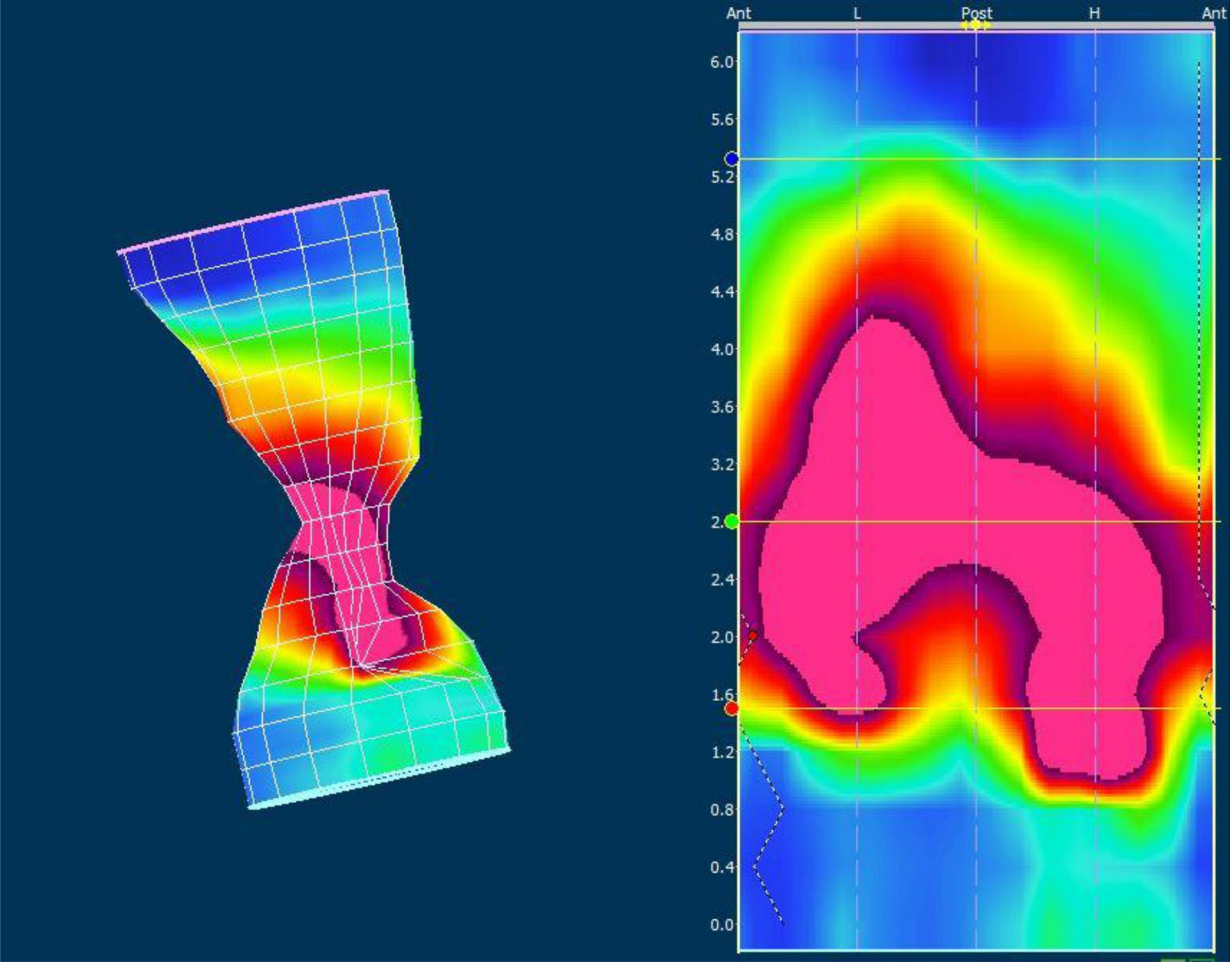
Pressure profile obtained by HRAM showing a lambda pattern during squeeze maneuver. On the right a normal characteristic λ-pattern after 2D opening of the 3D pressure cylinder on the left. Numbers indicates distance in length from anal verge in centimeters. *Ant* anterior, *Post* posterior, *L* left, *H* right. Numbers indicates length in centimeters.

After introducing the probe, a resting period of one minute was awaited before measurements. The resting pressure was measured continuously during a period of 20 s and repeated three times. The mean value for these three measurements was used for the calculation. Squeeze pressure and push maneuvers were then performed three times with 30 s intervals and the mean value was used for the calculation. The presence of a lambda pattern was registered on the 2D opening of the 3D pressure cylinder during the squeeze maneuver indicating normal function of the puborectalis muscle and external anal sphincter (Fig. 1). This is observed as maximal pressures in the posterior part of the proximal high-pressure zone (HPZ) which is corresponding to the puborectalis muscle and the anterior part of the distal HPZ which is corresponding to the external anal sphincter. In order to elicit the recto-anal inhibitory reflex (RAIR) the rectal balloon was forcefully inflated with increasing volumes of air in 10 ml aliquots stopped at 60 ml if the reflex was not elicited. Rectal sensitivity was examined by consecutively inflating aliquots of 10 ml of air until first sensation, urge to defecate and discomfort was registered. The procedure was stopped at a volume of 400 ml. The HPZ area was calculated as (rectal pressure + (anal resting pressure – rectal pressure)) × 0.25)^30^. Pressure measurements were all referenced to actual atmospheric pressure.

**Figure 2:**
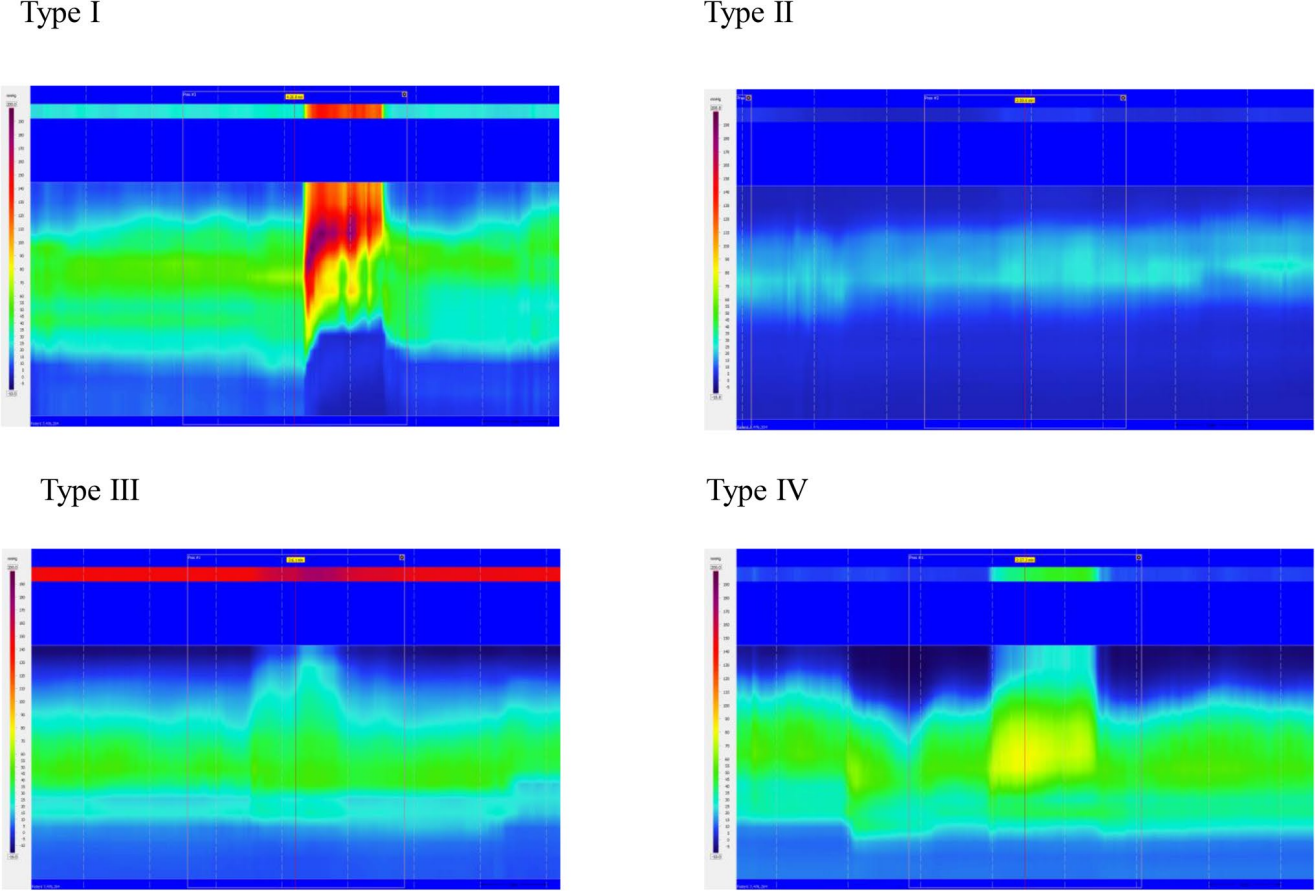
Dyssynergic patterns from HRAM during attempted defecation. Tracings from high resolution anorectal manometry, revealing four different patterns during attempted defecation in study patients. In type I dyssynergia the patient generates an adequate propulsive force (rise in intra-rectal pressure > 40 mm Hg) accompanied by a paradoxical increase in the anal sphincter pressure. In type II dyssynergia the patient is unable to generate an adequate compulsive force with a paradoxical small increase in anal sphincter pressure. In type III dyssynergia the propulsive rectal force is adequate but no relaxation of the anal sphincter. In type IV dyssynergia generated propulsive force is insufficient with inadequate relaxation of the anal sphincter.

Dyssynergic defecation patterns were identified on HRAM-tracings during attempted defecation. They were divided in to either type I, II, III or IV (Fig. 2). If one of the three attempted defecations were without signs of dyssynergic defecation pattern the defecation pattern was considered normal.

The CCCS was correlated to findings from colonic transit time (CTT) and HRAM.

In patients fulfilling the diagnostic criteria for functional chronic constipation (Rome IV criteria), DD was defined according to the following diagnostic criteria^31^.

A. Patients must demonstrate an obstructive pattern of defecation during high resolution anorectal manometry at more than one attempt
B. A prolonged colonic transit time

Dyssynergic pattern was based upon the results from the HRAM , and DD was based on the clinical criteria. Hence, it is possible to present a dyssynergic defecation pattern at HRAM without fulfilling the criteria for DD.

**Figure 3:**
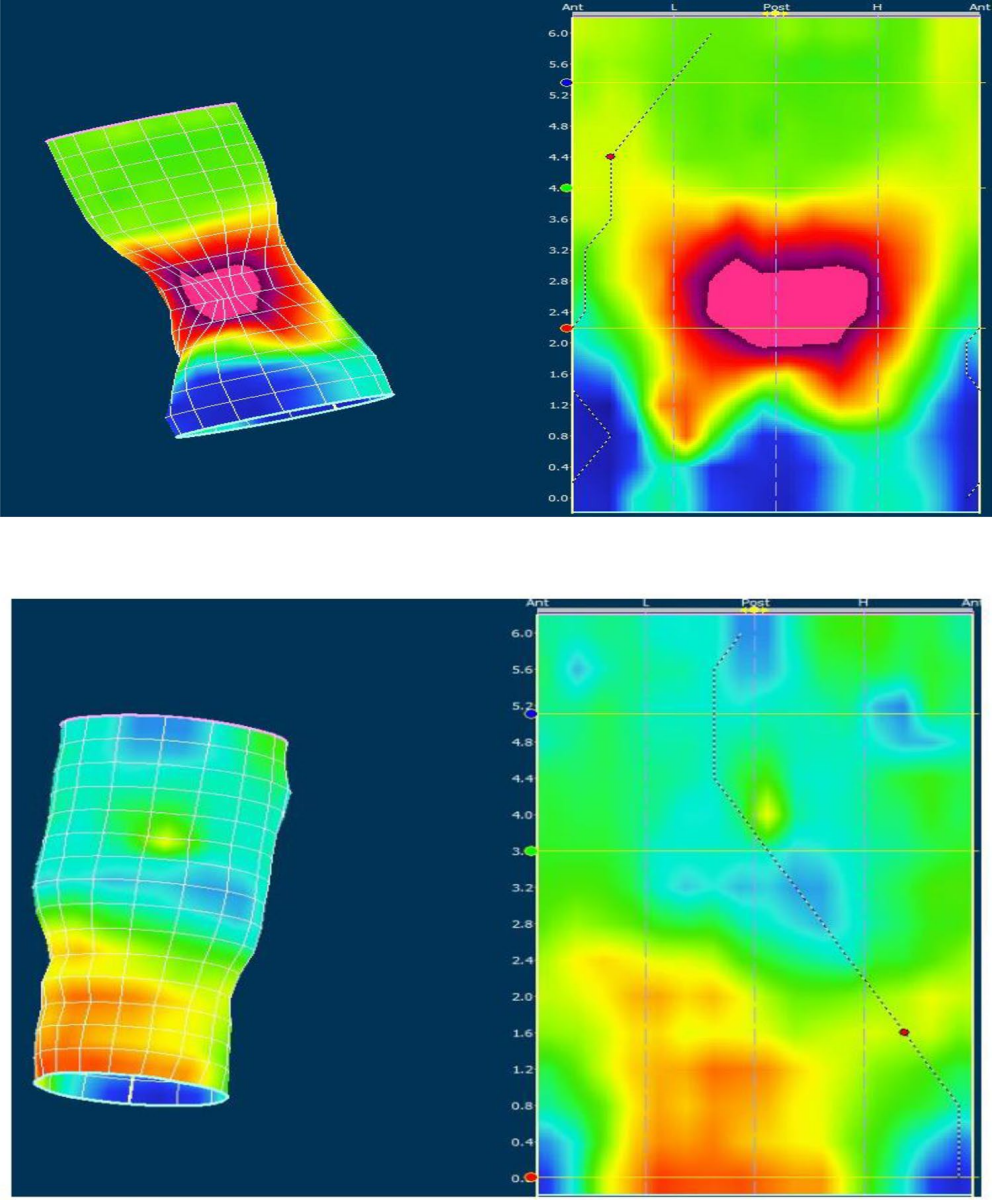
Individual muscle involvement during attempted defecation. High resolution anorectal manometry results in two constipated patient during attempted defecation. High pressure areas are seen with “warm” colors (red/purple) and low pressure areas with “cold” color (blue/green/yellow). On the left is presented a three-dimensional cylindrical pressure-distribution during attempted defecation. A two-dimensional landscape plot of the cylindrical presentation is presented on the right. In the upper picture a high pressure area is located in the posterior and proximal part of the anal canal indicative of paradoxical activation of the puborectalis muscle. In the lower picture a high pressure zone is observed in lower part of the anal canal interpreted as external anal sphincter contraction. Numbers indicates length in centimeters. *Ant* anterior, *Post* posterior, *L* left, *H* right.

Moreover we assessed the individual muscle activation during attempted defecation on three-dimensional cylindrical pressure-distribution plots as a localized pressure increment (Fig. 3). If the pressure increment was localized to the posterior and proximal part of the anal canal it was attributed to the puborectalis muscle. Pressure increment in the distal part of the anal canal was attributed to the external anal sphincter.

Each manometry data set was assessed by the same author (TBM) with more than five year experience in interpreting data from HRAM. Data was assessed with an interval of 2 weeks and if discrepancies appeared, data was assessed again after another 2 weeks at which intra-observer agreement on all data was achieved.

#### Colonic transit time test

Patients were asked to take a break from laxatives one week before the first day of capsule ingestion. One gelatin capsule was ingested on six consecutive days at nine a.m. Each capsule contained 10 radio-opaque poly-urethrane markers containing 40% barium sulphate (P.A. Mauch, Münchenstein, Switzerland). Shape of the markers varied from day one to day six containing; rods, spheres, large rings, cubes, small rings and rods respectively. A plain X-ray of the abdomen was performed on day seven without any bowel preparation and if possible during the same follow-up appointment as for HRAM^32^. In some instances it was not possible to coordinate different examinations on the same day.

**Figure 4:**
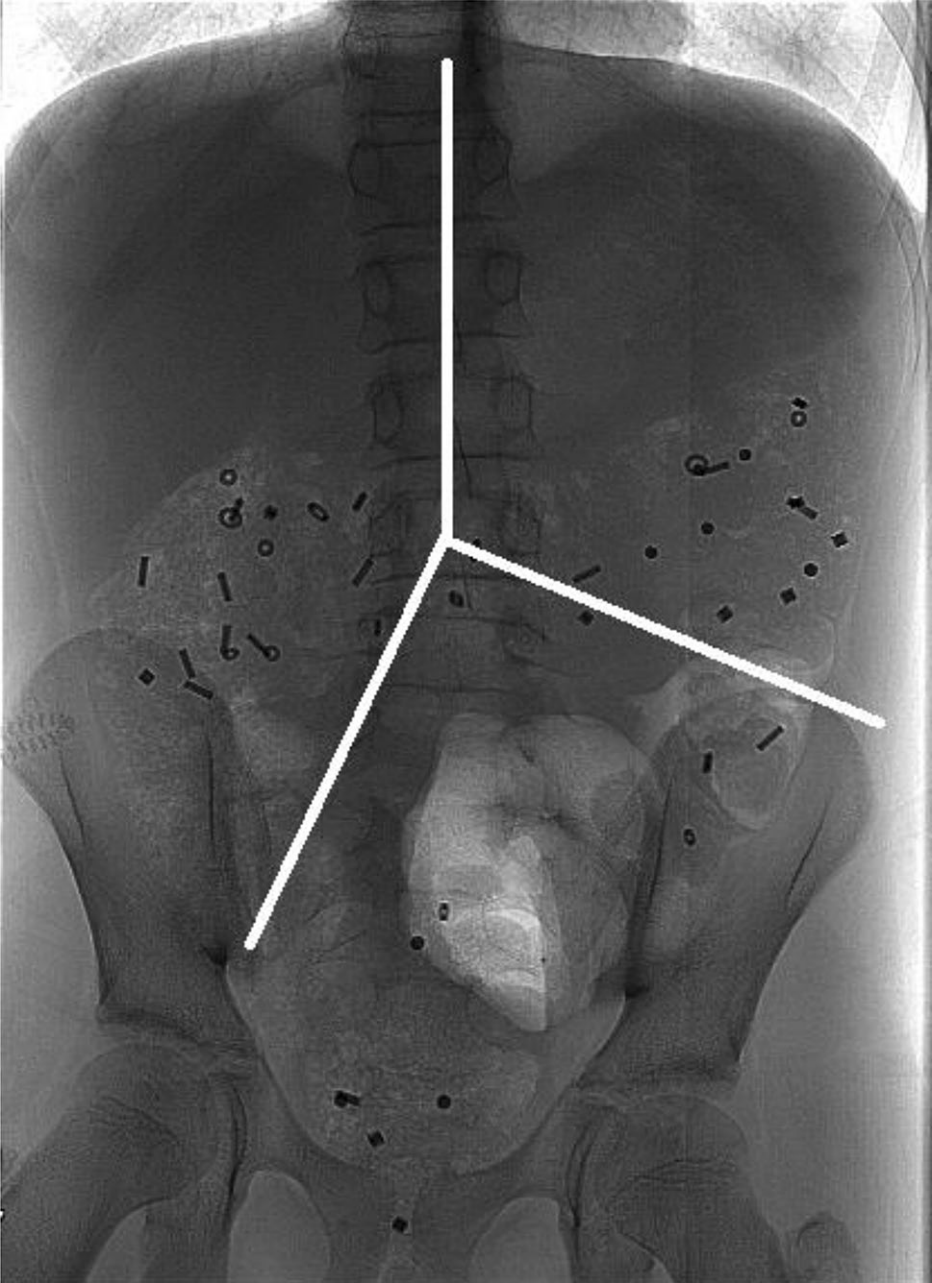
Abdominal frontal x-ray with colonic segments. In this frontal x-ray containing radio-opaque markers, the imaginary white lines mark the areas for segmental colon transit times. From the center of vertebrae L5, one line runs cranially along the vertebrae. The two other strait lines run towards the left iliac crest and the right pelvic outlet. The formed upper right area, the upper left and the lower left areas represents the right colon, the left colon and the recto-sigmoid colon respectively.

For interpretation of the X-rays three imagine lines were placed from the center of the fifth lumbar vertebrae. One line was placed cranially along the vertebrae, one line to the right pelvic outlet and one line towards the left iliac crest (Fig. 4).

Total numbers of radio-opaque markers were counted by a radiologist and by the first author of this manuscript (TBM). If discrepancies between numbers of counted markers occurred, an agreement on number of markers between radiologist and TBM was achieved. In addition to the total number of markers the number of markers was registered in the right colon, the left colon and the recto-sigmoid colon. With 10 markers per day each marker is equivalent to 0.1 days = 2.4 h. Therefore the formula M × 2.4 where M is sum of markers can be used on both the total and segmental colonic transit times providing transit times in hours.

Normal upper limit for total colon transit time was set to < / = 70 h for women and < / = 60 h for men^33^.

### Ethics

The study was conducted according to the 7th revision of the Helsinki declaration (2013). Verbal and written informed consent was obtained from adult participants and in participants below 18 year of age, from parent(s) or guardian(s). The study was approved by the National Committee of Health Research Ethics (S-20140017) and the Danish Data Protection Agency. It was registered in Clinical.Trials.gov. (NCT02624232).
